# Heatwave duration, intensity and timing as drivers of performance in larvae of a marine invertebrate

**DOI:** 10.1038/s41598-025-98259-7

**Published:** 2025-05-07

**Authors:** Margot Deschamps, Luis Giménez, Charlotte Astley, Maarten Boersma, Gabriela Torres

**Affiliations:** 1 Alfred-Wegener-Institut, Helmholtz-Zentrum Für Polar- und Meeresforschung, Biologische Anstalt Helgoland, Helgoland, Germany; 2 Ifremer, HMMN, Unité Halieutique Manche Mer du Nord, Laboratoire Ressources Halieutiques, F-62200 Boulogne-sur-Mer, France; 3 School of Ocean Sciences, Bangor University, Menai Bridge, UK; 4 Department of Environmental Science, Barnard College, Columbia University, New York, USA; 5 Alfred-Wegener-Institut, Helmholtz-Zentrum Für Polar- Und Meeresforschung, Wattenmeerstation Sylt, Sylt, Germany; 6 University of Bremen, Bremen, Germany

**Keywords:** *Carcinus maenas*, Global warming, Larval performance, Marine heatwaves, Climate-change ecology, Animal physiology

## Abstract

In marine ecosystems, crustaceans face an alarming threat from the increasing frequency and intensity of marine heatwaves as their early planktonic stages are particularly temperature sensitive. While the impact of heatwaves on adult crustaceans is well-studied, their effects on larvae remain underexplored. This study focuses on heatwave effects on larvae of the European shore crab, *Carcinus maenas*. Through a factorial experiment, larvae were exposed to different heatwaves of varying onset timings, durations, and intensities. Survival, development duration, and dry mass decreased under intense heatwaves, with more severe effects observed when heatwaves occurred later in development, highlighting a stage-specific sensitivity to heatwave. We also identified a “region of existence” beyond which larval performance was compromised compared to baseline temperatures. This region defines the heatwave components considered “extreme” for the organism, as well as those inducing neutral or positive effects on performance. Additionally, we distinguished heatwave effects (characterised by their components) from those attributed to the average temperature experienced during the experiments. Our findings demonstrated that larval performance was lower during intense heatwaves compared to the performance expected under a constant average temperature. These findings emphasize the importance of considering heatwave timing relative to the life cycle for predicting marine population responses to climate change.

**Supplementary Information:**

The online version contains supplementary material available at 10.1038/s41598-025-98259-7.

## Introduction

Anthropogenic climate change is a significant threat to biodiversity worldwide^1^. Alongside the ongoing trend of warming, discrete extreme weather events, such as heatwaves, are becoming more frequent and intense^2–5^. Studies on the biological impacts of heatwaves on organisms have demonstrated that extreme events can impact biological processes at all levels, from the individual to the ecosystem^6–8^. Consequently, it is crucial to understand and predict how organisms, particularly those with short generation times and high sensitivity to temperature changes, respond to specific events^9–11^.

In marine ecosystems, extreme seawater temperature events have been documented around the world, sometimes exhibiting temperature 3–4 °C higher than the temperature typically observed on those dates^12–15^. Following the definition by Hobday et al., (2016)^16^, there is a consensus that when these events exceed the 90th percentile of a climatological baseline and last more than five days, they are classified as marine heatwaves. In recent years, growing ecological and socioeconomic concerns about impacts of marine heatwaves have led to increased research efforts aiming at defining, categorising, and exploring their consequences across different levels of biological organization^8^. These impacts can vary widely, ranging from positive to negative, depending on the organism studied but also on the seasonal timing of the heatwave^17–19^. At the individual level, increased basal metabolic rates and energy demands induced by marine heatwaves can influence the growth and reproduction of marine organisms^20–22^. In spring, when heatwave temperatures generally fall within the species thermal ranges, the effects will be mostly positive. However, these effects may become negative if temperatures exceed that range^8^, as is more likely with summer heatwaves^23^. Population-wise, responses to marine heatwave can range from recruitment failure through reproductive failures or mass mortality events, ultimately threatening species persistence^24,25^, to range expansion, increased abundance and reproduction^8^. Lastly, heatwaves might trigger shifts in marine community structure, favouring warm-water species over those adapted to cooler waters^26,27^.

The predicted increase in frequency and magnitude of heatwaves^4,5^ has become particularly alarming for marine organisms with complex life cycles, which comprise the great majority of marine invertebrates and fish. Some of these organisms such as crabs, oysters and sea urchins develop through a larval phase drifting in the water column, while the juvenile and adults live associated with the sea bottom. Larvae contribute to connectivity between populations^28^, range expansion^29–31^, and can drive recovery of local populations from mass mortalities^32^. Moreover, larval stages are often more sensitive to temperature increases and exhibit a narrow subset of the species thermal niche compared to their juvenile and adult counterparts^33^. Therefore, depending on the heatwave season, larvae can be impacted by smaller temperature increases compared to adults, which may affect the recruitment success, dispersion, and survival^34^.

While the impact of warming on adult marine invertebrates is well-documented, a proper understanding of the effects of marine heatwaves on larval performance is currently missing^35,36^. Field investigations examining the impacts of past heatwaves on larval assemblages are limited to a few studies^37,38^. Moreover, while marine heatwave simulations and mesocosm experiments were carried out (Meunier et al., *in press*), few studies have focused on understanding the effects on larval stages^39–41^. Furthermore, these studies often concentrate on marine heatwaves intensity, overlooking the cumulative effects of various components such as duration and timing. Additionally, the common practice of measuring heatwave impacts against a constant baseline temperature does not adequately separate the effect of the acute temperature exposure inherent to the heatwaves from the effect of experiencing an increased average (but constant) temperature. Importantly, a growing body of literature shows that biological responses to fluctuating environments differ from those occurring under constant conditions^42–44^. Unlike exposure to a constant temperature, changing environments can drive phenotypic plastic responses^45^. However, in the context of heatwaves, the timescales at which an organism operates (e.g., generation time, development duration) must be considered in relation to the heatwave timescales^46,47^ (i.e., timing and duration) as this relationship will determine whether phenotypic plasticity can occur. Additionally, because of the sensitivity of metabolic rates to temperature, heatwaves have the potential to increase developmental rates, hence modifying the way they are experienced by organisms^47^. Therefore, there is a need to differentiate the impacts of the heatwave components, and to determine the conditions where the effect of heatwaves differs from those expected by experiencing an increased but constant temperature.

**Fig. 1:**
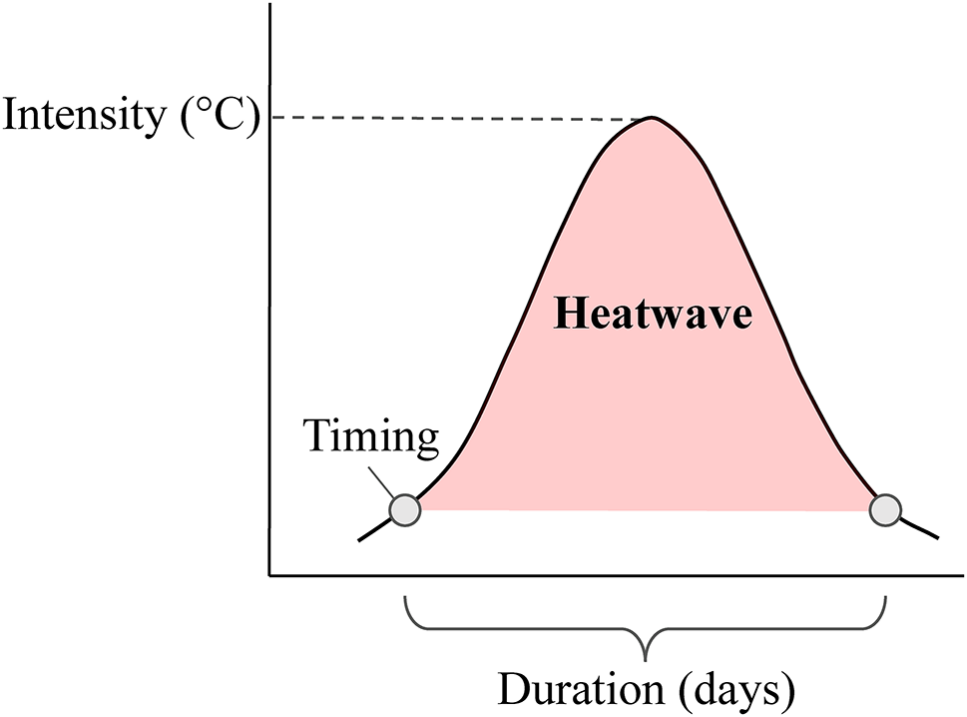
Heatwave components diagram. Intensity, timing and duration represent the maximum intensity (°C), the time of the start and the duration (days) of the event, respectively.

Here, we quantified the role of three primary components of marine heatwaves (i.e., intensity, timing, duration) (Fig. 1) on larval performance (survival, developmental time, and body mass at metamorphosis) through laboratory experiments, exposing larvae of the shore crab *Carcinus maenas* to warm events of different intensity, duration, and timing. To achieve this objective, we conducted two experiments. The first experiment manipulated the timing and intensity of the warm events while keeping the duration constant (i.e., 10 days, corresponding to the duration of heatwaves frequently detected in the North Sea^48^). In the second experiment, larvae were exposed to warm events of different intensity starting at different times during their development and ending after metamorphosis, with no recovery period post warm event to simulate conditions where larvae are exposed to late summer heatwaves, common in the study area^48^. In the North Sea, *C. maenas* larvae are currently living close to their thermal optimum during summer (15–18 °C)^49,50^. As a result, they only have a safety margin of a few °C before negative effects occur. Therefore, we hypothesize that increasing the intensity of the event should reduce larval performance as temperatures approach critical thermal limits. We also believe that the effects of timing would point towards stage-dependent thermal tolerance with potential increased sensitivity at the time of metamorphosis^51–53^. Additionally, we disentangled the effect of warm events from the effect of warming by determining the combinations of the components producing a true warm event effect (i.e., where responses differ from those occurring if larvae are exposed to the average temperature experienced during the experiments). Here, larvae were exposed to constant temperatures, and we then compared expectations obtained from such experiments with observations from experiments exposing larvae to warm events.

## Materials and methods

The European shore crab *Carcinus maenas* was used as a model system. *C. maenas* is an omnivorous crab, playing an important role in benthic ecosystems, with a native distribution over much of the European coast^54,55^. *C. maenas* is also a global invader^54^, well established, for instance, on the coasts of North America^31^. Larvae develop through four zoeal stages, followed by a metamorphosis to megalopa that recolonises (= settles on) the juvenile-adult habitat^49,56^.

### Animal collection and husbandry

Larvae were obtained from *C. maenas* berried females, collected in June 2023 during their reproductive period on the island of Helgoland (German Bight, North Sea: 54° 10′53″, N 7°53′10″ E) and transported to the laboratory at the Marine Station on Helgoland^57^. Each berried female was maintained individually in a 5 L aquarium filled with aerated UV-treated filtered seawater (2 μm, salinity 32.5). Aquaria were kept in a temperature-controlled room at 18 °C ± 0.5 °C with a 12:12 light: dark cycle. Females were fed twice a week, and water was changed daily to ensure high water quality during hatching. Freshly hatched larvae were separated from the females and distributed randomly in groups of 10 individuals per beaker (60 ml each). Each beaker was then randomly attributed to a treatment. Larvae were reared in natural UV-treated filtered (2 μm) seawater and fed daily with *ad libitum* freshly hatched *Artemia* sp. nauplii. To ensure high water quality, water was changed daily before the food was renewed (see^58^ for larval rearing procedures).

### Experimental design

When considering experimental approaches to study heatwave effects on marine organisms, different methodologies emerge^39,47,59^. First, one can simulate realistic heatwaves incorporating natural temperature variations. This approach enables a detailed examination of how specific heatwave conditions impact larval development but lacks mechanistic understanding and predictive capacity for different heatwave scenarios not directly studied in the experiment^44^. Second, adopting a mechanistic approach involves using idealized heatwave conditions that are typically different from what is experienced in the field (e.g., faster temperature increase rate). While this method involves limited realism^59^, it enhances the understanding and predictive ability regarding the impacts of future heatwaves and lays the groundwork for subsequent studies considering realistic heatwaves. In this study we opted for the second approach although for semantic reasons we refrained to use the term “heatwave” to describe the idealized heatwave simulated here. Indeed, the term heatwaves is already well-defined in the literature for field observations^16^ and we do not wish to imply that our idealized heatwaves are equivalent to actual heatwaves. We will use the term “warm pulse” and “warm press” to refer to the first and second experiment respectively, and “warm event” when referring to both experiments. In ecology, the term “pulse” is commonly used to describe a disturbance that is typically intense but short-lived, with conditions returning to their former level afterward^60^. This concept has been applied to extreme events such as flooding, drought, and heatwaves^6,61^. In contrast, the term press defines a disturbance that once started continues to occur^60^.

Experiments were conducted in temperature-controlled rooms, with three replicate beakers per treatment combination. The experiment was repeated four times, using larvae from a different female for each repetition, in order to account for potential effects associated with parental effects.

**Fig. 2:**
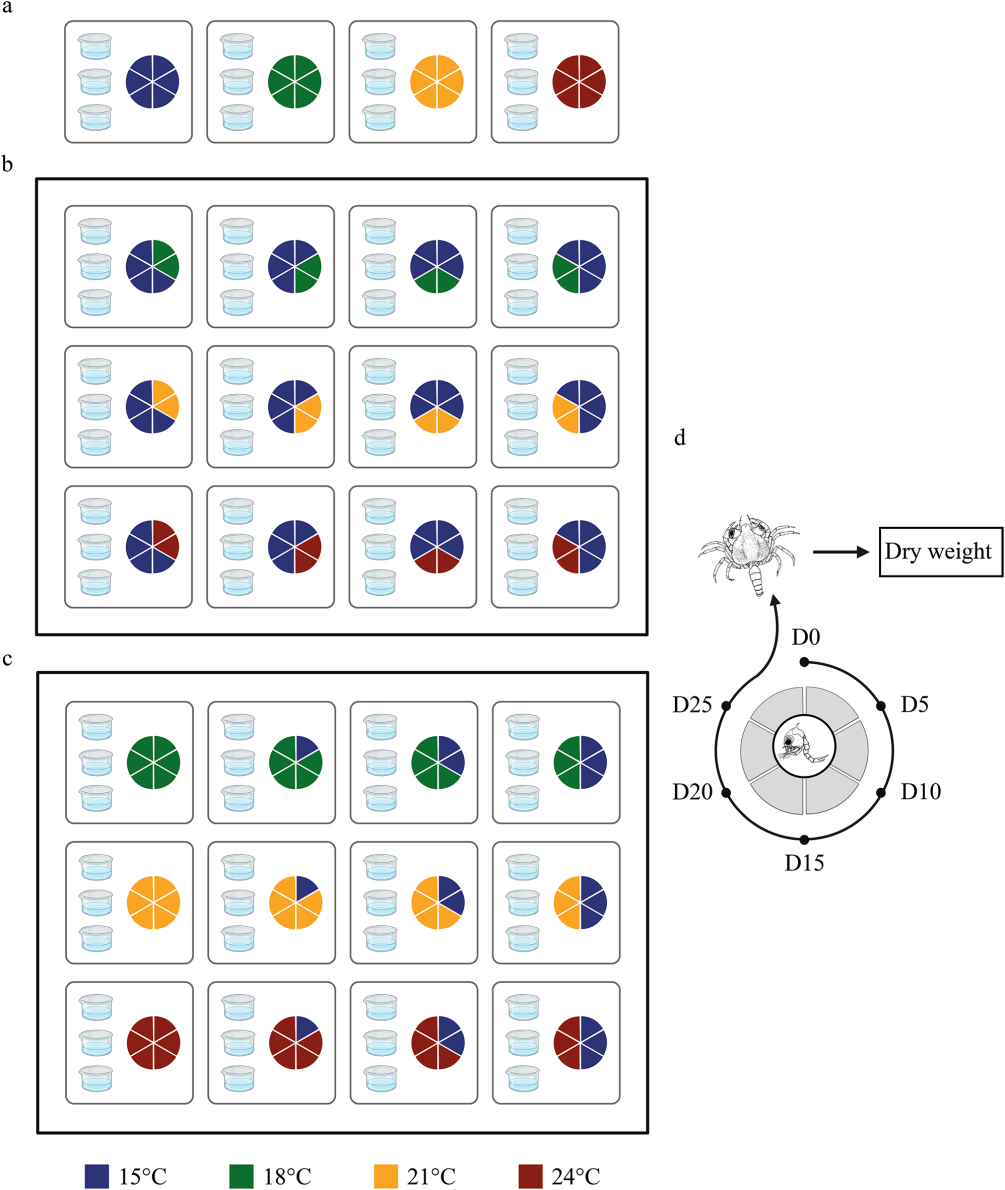
Experimental design for warm event simulations. Larvae were reared in groups of 10 individuals each in three replicate beakers representing each combination of the above mention factorial design. Pie charts symbolise the complete zoeal life cycle of *C. maenas*, with each sector corresponding to 5 days of development. (**a**) Four controls were carried out wherein larvae were maintained at constant temperatures (15, 18, 21 & 24 °C) throughout their larval cycle until metamorphosis. (**b**) Warm pulses experiment. Larvae were kept at the baseline temperature (15 °C) and exposed for 10 days to higher temperatures (18, 21 & 24 °C) starting on days 0, 5, 10 and 15. (**c**) Warm presses experiment. Larvae were reared at the baseline temperature (15 °C) and then kept at higher temperatures (18, 21 & 24 °C) starting on days 0, 5, 10 and 15 until they reached megalopa. Note that days 0 for the warm press experiment are the same treatment as the control. (**d**) Larval cycle of *C. maenas*. Zoea were reared until they reached megalopa. At metamorphosis (within 24 h of moulting to megalopa), individual megalopa were sampled for further analysis of dry mass.

To differentiate the specific effect of warm events from those attributed to different temperatures, four groups of larvae (control groups) were reared at constant temperatures (15, 18, 21, and 24 °C) from hatching until metamorphosis to the megalopa. This was done using three replicate rearing beakers × four control temperatures × four females (total = 48 beakers; Fig. 2a).

#### Warm pulse

A full factorial design was carried out manipulating the intensity and timing (i.e., time of the start of the exposure) of a 10-day warm pulse (Fig. 2b). Here, we were interested in determining the effects of heatwaves of time scales shorter than the duration of the larval phase. The effects of those heatwaves are less studied in the literature, but they are often more frequent than prolonged heatwaves of several weeks to months of duration^5^. Larvae were reared from hatching at a constant seawater temperature (15 °C), representing the average water column temperature in June^62^ when the larvae start to hatch. This baseline temperature differed from the temperature at which females were maintained (i.e., 18 °C) because, in June, the temperature in the intertidal, where the embryos develop is higher than the temperature of the seawater on the open coast. This distinction highlights the difference between the natural hatching conditions and the environmental baseline temperature used in our analysis.

To simulate warm pulses, larvae were exposed to elevated temperatures of 18, 21, and 24 °C. According to the marine heatwave definition by Hobday et al., (2016)^16^, the threshold (Q90) for classifying an event as a marine heatwave in the North Sea during summer corresponds to a temperature increase of approximately + 1 °C above the climatology (Deschamps pers. Obs.). Therefore, the temperature range studied here can be considered as marine heatwaves. The exposure to elevated temperatures started on days 0, 5, 10, and 15 after hatching (i.e., timing) and lasted for 10 days. Before and after the exposure to higher intensities, larvae were kept at the baseline temperature of 15 °C, and continued their development until they reached the megalopa stage. Overall, this design used three replicates rearing beakers × three intensity levels × four timings × four females (total = 144 beakers; Fig. 2b).

#### Warm press

This design simulated a situation where individuals would encounter heatwaves at different times of the larval phase and then experience this temperature until metamorphosis. Such scenario would occur if larvae were hatching at different times with respect to the initiation of a summer heatwave. Larvae were again exposed to the same intensities and timing as in the previous experiments. However, the elevated temperatures lasted until larvae metamorphosed to the megalopa (Fig. 2c). Hence, in this experiment, the timing of the warm press covaried with their duration, i.e., earlier timing resulted in longer warm presses. This design consisted of three replicate rearing beakers × three intensity levels × four timings × four females (total = 144 beakers; Fig. 2c). From this experiment the 48 beakers from t_0_ are the same as the control constant temperature. Larvae of each different female were treated in separate experiments, so that at any given time we handled 36 (warm pulses) + 36 (warm presses + control) + 3 (15 °C constant) beakers.

Larval performance was quantified as survival, development duration, instantaneous growth rate of megalopa, and body mass of the megalopa. During the daily water change, survival was recorded through visual inspection, and development duration was determined by counting moults and visual assessment of larval size. To determine the dry mass, recently moulted megalopa (within 24 h of metamorphosis) were transferred from the beaker onto a filter and gently rinsed with distilled water. Excess of water was removed using lint-free wipes (Kimtech Science™ Precision Wipes), and individual megalopa were placed into pre-weighted tin cartridges before being stored at − 20 °C. Samples were then freeze-dried for 24 h (Christ Alpha 1–4 freeze dryer) and dry mass was determined using a microbalance (Sartorius Cubis^®^ MCA2.7 S, ± 1 µg accuracy). We also determined the body mass of freshly hatched larvae (same procedure as for megalopa), in five replicates per female of origin, consisting of 50 larvae per replicate.

### Data analysis

All statistical analyses were conducted under R environment (R Core Team, 2020) with a threshold of significance set at 5%.

Survival was calculated as the proportion of larvae surviving from hatching to each stage. Proportions were then transformed into the logistic scale. Because the logistic scale is defined for real numbers only, proportions were transformed before the analysis using the formula:

$$\:\text{P}=\:\frac{\text{p}\left(\text{N}-1\right)+0.5}{\text{N}}$$

Where **p** is the proportion of survivors and **N** is the initial number of larvae assigned to each replicate (i.e., 10). Development duration to zoea II, III, IV, and megalopa was calculated as the time elapsed (in days) from hatching to the selected stage. The instantaneous growth rate was calculated using the formula:

$$\:\frac{\text{l}\text{o}\text{g}\left(\raisebox{1ex}{${\text{D}\text{W}}_{\text{M}}$}\!\left/\:\!\raisebox{-1ex}{${\text{D}\text{W}}_{\text{Z}\text{I}}$}\right.\right)}{\text{D}}$$

Where **DW**_**M**_ is the megalopa dry mass, **DW**_**ZI**_ is the freshly hatched zoea I dry mass and **D** is the time elapsed from hatching to metamorphosis to megalopa.

To evaluate the warm event effects on survival, development duration, and growth (i.e., dry mass, instantaneous growth rate), linear mixed effect models (LMMs) were performed separately. All model assumptions were verified by checking Residuals and Overdispersion using the *DHARMa* package^63^. To assess model fit, model selection procedure was used based on the corrected Akaike Information Criterion (AICc)^64^. Specifically, the best-fitting model was determined by identifying the one with the lowest AICc value, with an exception made when the ΔAICc (i.e., difference between a candidate model and the model with the lowest AICc) was ≤ 3. In such case, we compared both models using likelihood ratio tests (LRT). If models differed significantly, the lowest AICc model was selected. Otherwise, the model with the lowest number of parameters was chosen. If the diagnostic tests indicated that the residuals or dispersion violated model assumptions, the closest model in terms of AICc was chosen. In the first step, model selection based on Restricted Maximum Likelihood (REML) was initially performed to select the best random structure of the model. Because experiments were repeated four times with four different females, the female of origin (♀) was consistently included as a random factor, regardless of whether the AICc for a model without random terms was lower than any other model. In the second step, the model with the best random structure was refitted with Maximum Likelihood (ML), and subsequent model selection was carried out to determine the fixed factors using ML. For all response variables, the full mixed model included the fixed factor temperature intensity in interaction with the timing of the warm event.

**Fig. 3:**
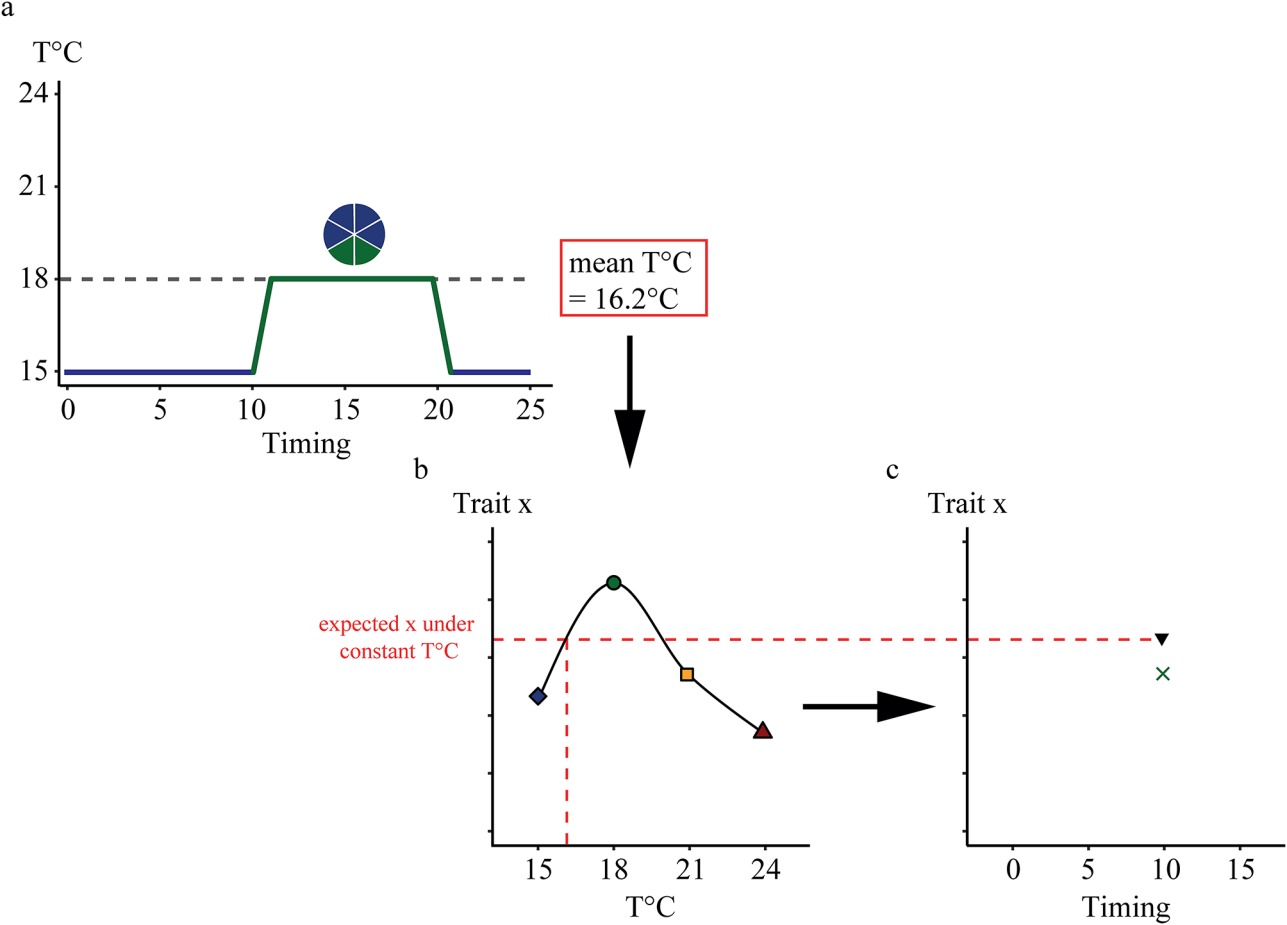
Workflow for predicting larval performance values (survival, development duration, and growth traits) under the average temperature experienced during warm event experiments. (**a**) The average temperature experienced by the larvae if the temperature was constant throughout the experiment was calculated for each replicate across the different warm event conditions. (**b**) Expected trait values were obtained by fitting models with the control temperatures (15 °C filled blue diamond, 18 °C filled green circle, 21 °C filled yellow rectangle and 24 °C filled red triangle) as fixed effects and the individual females (♀) as a random effect. (**c)** The expected trait values (filled inverted triangle) under the average temperature were compared with the observed values during warm events (**×**) by fitting LMMs. The fixed factor, expected/observed was included in the model, in interaction with temperature and timing. The ♀ was used as a random factor.

An important objective was to determine if the responses to the warm events (characterised by their components) would differ from the response attributed to the average temperature experienced during each experiment (Fig. 3). We compared expectations from the average responses with observations as follows: First, we calculated the average temperature over the warm event experiments. For example, if an individual larva developed over 25 days, spending 15 days at 15 °C and 10 days at 18 °C, the average temperature experienced would be 16.2 °C (Fig. 3a). Second, we used this value to predict the performance (survival, development duration, growth traits) using the statistical functions fitted to the effect of constant temperature (control treatment experiment) on each performance indicator (Fig. 3), always keeping ♀ in the random structure. Here, we computed the expectations of the LMMs using the function “predict”. For growth rates, we could not find a simple function fitting the response over the full temperature range; instead, we fitted separate polynomials in the log-transformed growth rates in the range 15–21 °C and a linear model for 21–24 °C. Each model was then used separately depending on the average temperature used to make expectations: the second model was specifically applied to predict instantaneous growth rates for scenarios involving 24 °C warm presses, starting either on day 0 (with a mean temperature of 24 °C) or day 5 (with mean temperatures ranging from 21.2 °C to 21.7 °C). Third, to assess the difference between the expected larval performance under average temperature experienced and those observed during warm event, a second LMM was performed. Here, a fixed factor, expected/observed (**EO**, categorical with 2 levels: expected and observed values), was included in the model in interaction with temperature intensity and timing (female of origin was used as a random factor). When a significant effect was observed, multilevel pairwise comparisons with a Tukey *p*-value adjustment were carried out posteriori. In some cases (i.e., instantaneous growth rates, see results), variance heterogeneity violated the assumption of homoscedasticity. Therefore, a variance heterogeneity term was added to the variance structure using the varIdent constructor function (R Package nlme); here, we modelled variance heterogeneity between the expected vs. observed groups. For the warm press experiment, it is important to emphasize that the observed and expected responses will coincide at t_0_ (= time of hatching) as a warm press starting at t_0_ corresponds to treatments where temperature was kept constant over the whole larval phase. Consequently, the absence of a significant difference between t_0_ observation and prediction validates the accuracy of the model in predicting performance.

To illustrate the comparison between warm event conditions and the early summer baseline temperature (i.e., 15 °C), the differences between the performance observed after exposure to the baseline temperature and the one observed after exposure to the warm event conditions were calculated. This comparison was visualized in a heatmap, representing the space of changes defined by the warm event components (i.e., timing and intensity). Larval performance was quantified as survival rates to megalopa and fitness with fitness calculated as the product of survival and body mass at metamorphosis. A difference < 0 indicated a positive effect of the warm event on larval performance, while a difference > 0 indicated a negative effect. No difference represented a neutral effect of the warm event on larval performance.

## Results

An important point in the analysis was to determine if any effects of the treatments characterising warm events were observed before experiencing the increased temperature. We checked for these effects by looking at performance at the second, third, and fourth zoeal stages and did not find any evidence of treatment effects occurring before the larvae actually experienced the warm event (see Supplement Section I: Table S1, Figs. S1-S2). Instead, responses were observed either during the warm event or after the larvae returned to the baseline temperature.

### Warm pulses experiment

**Fig. 4:**
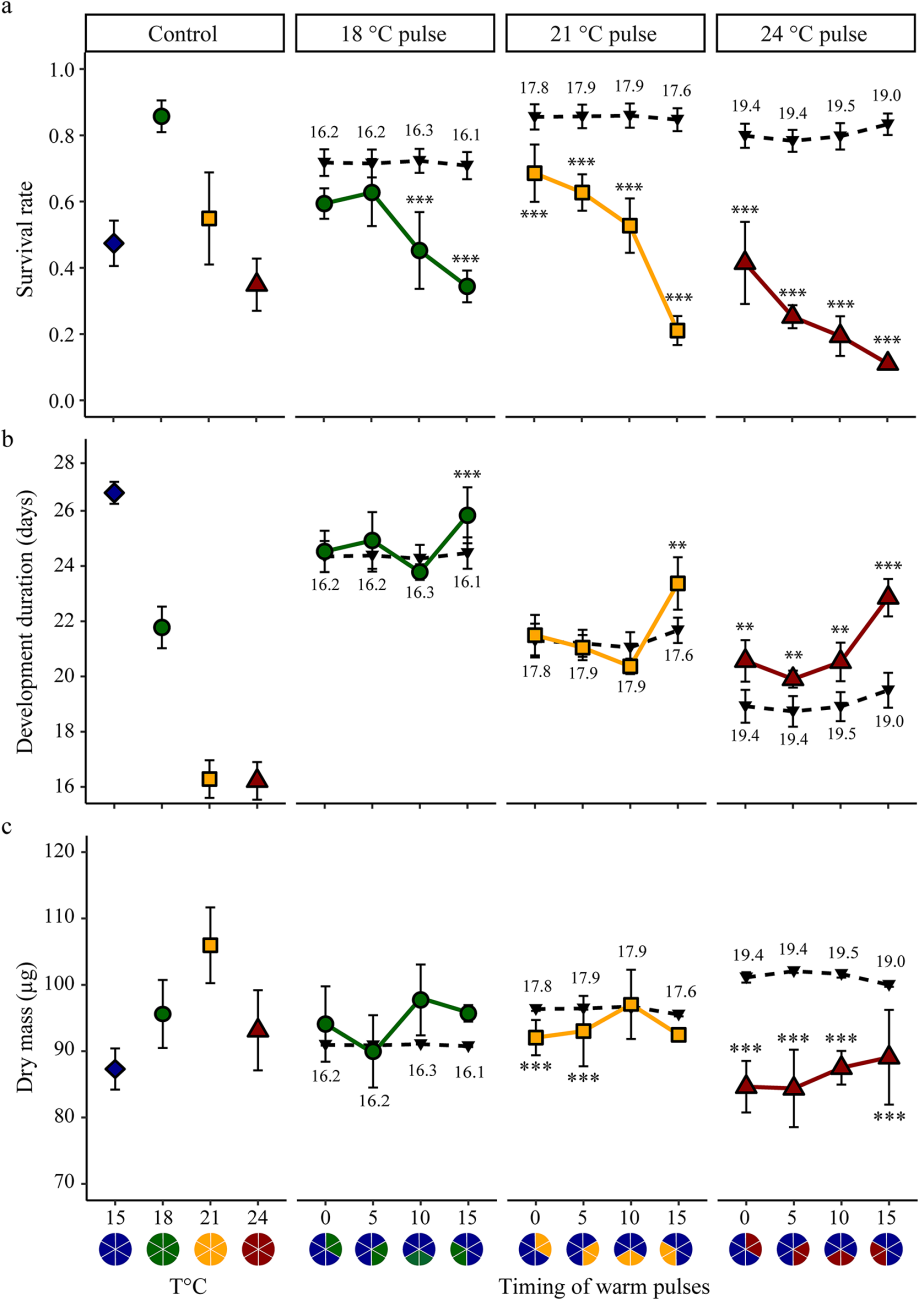
Survival rate, development duration, and dry mass after exposure to warm pulses. Comparison between observed (18 °C: filled green circle ; 21 °C: filled yellow rectangle ; 24 °C: filled red triangle warm pulses) and expected (filled inverted triangle) values under average temperature experienced throughout the experiment. (**a**) Survival rate to megalopa, (**b**) development duration from hatching to megalopa and (**c)** megalopa dry mass reared at control constant (left panel) or under warm pulse (right panels). Temperatures: 15 °C filled blue diamond, 18 °C filled green circle, 21 °C filled yellow rectangle and 24 °C filled red triangle. Each point represents the mean value ± standard error for each treatment per female (*n* = 4). Values above or below the black dotted line represent the average constant temperature experienced (°C) during the warm pulses. Asterisks indicate significant differences between expected and observed values for each treatment. *p* < 0.05*, *p* < 0.01**, *p* < 0.001***. Pie charts indicate control and warm pulse treatments.

The best models for survival and development duration (from hatching to megalopa) included the additive effect of intensity and timing (Fig. 4a; Table 1, S2). Increased intensity (i.e., 24 °C: Tukey-adjusted *p* < 0.001) resulted in reduced survival compared to low (i.e., 18 °C) and intermediate (i.e., 21 °C) intensities. Additionally, survival was significantly lower when the warm pulse started on day 15 compared to earlier timings. Survival under increased average (but constant) temperatures did not always match those observed under warm pulses (Table 1, S3). Specifically, survival rates at 18 °C pulses were lower than those expected for the corresponding average temperature, when the warm pulse started 10 days and 15 days after hatching. At the 21 and 24 °C pulse, survival rates were consistently lower than those expected under the average temperature, regardless of when the warm pulse occurred. The lowest survival rates were recorded during t_15_ warm pulse, with a significant drop to 34% at 18 °C, 21% at 21 °C, and 11% at 24 °C, in contrast to the survival rates of approximately 75, 86, and 80%, respectively, expected for the average temperature conditions.

**Table 1:** Warm pulse experiment: Best model selected for each variable of interest, based on AICc.

Variable	Best model	ΔAICc	2nd best model	Supplementary file
Survival rate	t + T°C	2	t × T°C	Table S2
Development duration	t + T°C	6	t × T°C	Table S2
Dry mass	t + T°C	6	t × T°C	Table S2
Instantaneous growth rate	t × T°C	0.5	t + T°C	Table S2
Predicted vs. expected survival rate	EO × t + EO × T°C + T°C × t	5	EO × T°C × t	Table S3
Expected vs. Observed development duration	EO × t + EO × T°C + T°C × t	3	EO × T°C + EO × t	Table S4
Expected vs. Observed dry mass	EO × t + EO × T°C + T°C × t	5	EO × T°C + T°C × t	Table S5
Expected vs. Observed instantaneous growth rate	EO × t + EO × T°C + T°C × t	2	EO × T°C + EO × t	Table S5

Factors include t: warm pulse timing, T°C: warm pulse intensity, EO: Expected vs. Observed value. ΔAICc: difference between the best model and the next closest model. Complete model selection tables are available in the supplementary file.

Increased intensity resulted in shorter development duration. Larvae had longer development at a lower intensity (i.e., 18 °C) as compared to those at intermediate and high intensities (Tukey-adjusted *p* < 0.001 in both cases) (Fig. 4b). Additionally, development duration was significantly longer when the warm pulse started at day 15 (compared to the earlier timings, i.e., t_0_; Tukey-adjusted *p* < 0.001). Again, the expectations for the average temperature experienced did not match the observed responses during the warm pulse experiment (Table 1, S4). The differences between expectations and observations were more pronounced at the highest intensity, where observations consistently exceeded the expectations, regardless of the timing (Fig. 4b). For intermediate and lower intensities, longer development duration was observed during t_15_ warm pulses as compared to the average temperature conditions. The largest difference between observed and expected development durations occurred during an intense pulse (i.e., 24 °C) starting at t_15_, where development duration was delayed by up to 3.5 days compared to the average temperature conditions. As larvae took 22.9 days to reach the megalopa stage under the warm pulse condition, development time increased by 15.3% during the intense pulse compared to the expectation.

Dry mass and instantaneous growth rates responded to both intensity and timing of the warm pulse (Figs. 4c, S3). The best model retained the additive effect for dry mass and the interactive effect for instantaneous growth rates (Table 1, S2). The intense pulse led to reduced dry mass (Tukey-adjusted *p* < 0.001 when compared to 18 and 21 °C) but the reduction was less pronounced when the warm pulse started on day 10 compared to those starting earlier (t_0_ vs. t_10_; Tukey-adjusted *p* < 0.05 and t_5_ vs. t_10_; Tukey-adjusted *p* < 0.001). The instantaneous growth rate increased with intensity, but the increment was smaller for later warm pulses (Fig. S3). Expectations from the average temperature experienced during the warm pulse differed from observations (Table 1, S5). Deviations from expectations for dry mass and instantaneous growth rates were stronger for the intense pulse, with reduction observed across all timing compared to the expectations (Fig. S3).

**Fig. 5:**
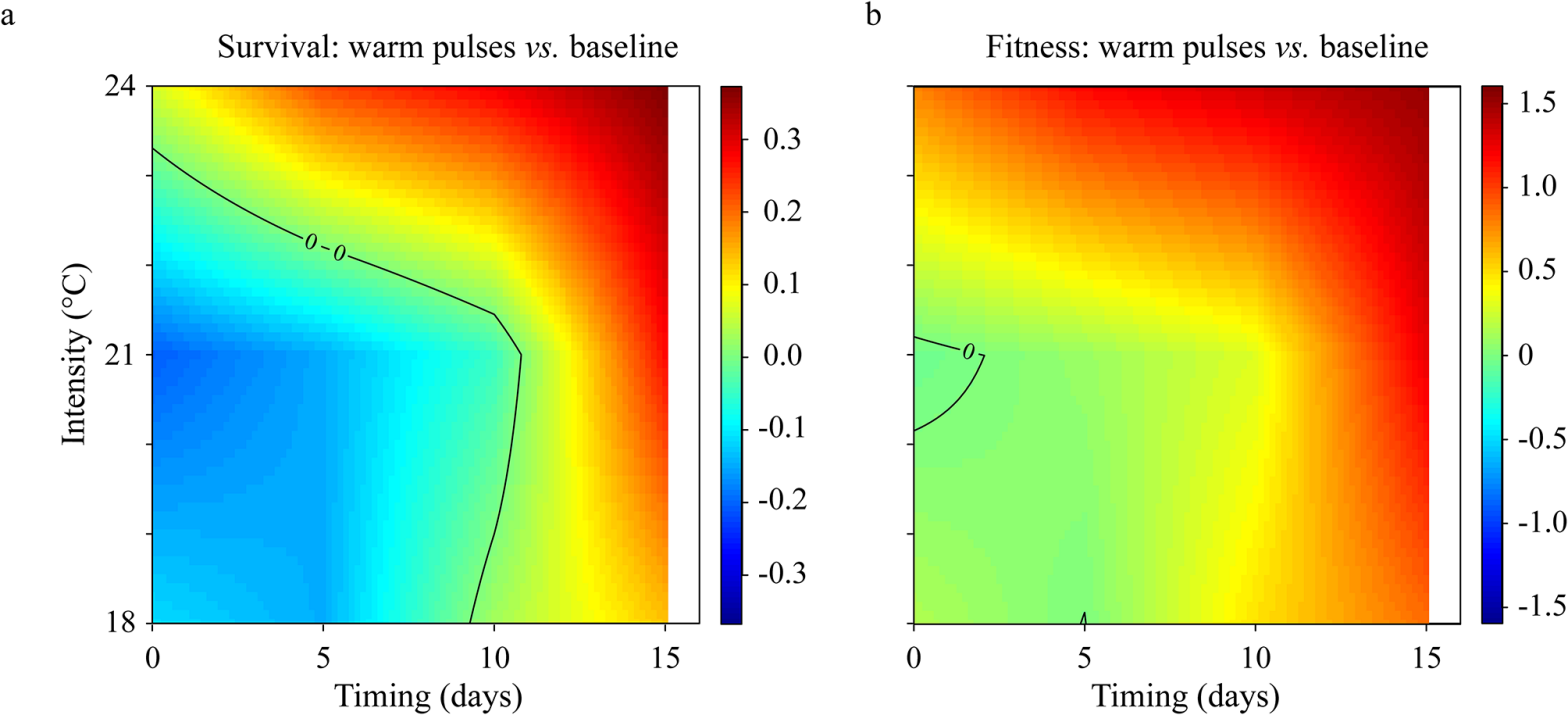
Difference in larval performance between the baseline constant temperature and warm pulse conditions. Differences in (**a**) larval survival and (**b**) fitness. Fitness is calculated as the total megalopa production (mg). Colour gradient represents the difference between response at the baseline temperature: 15 °C, and after exposure to warm pulses. Differences < 0 indicate positive effects of the warm pulses (i.e., the region below the “0” isoline). Differences > 0 indicate negative effects of warm pulses (i.e., the region extending beyond the “0” isoline). Differences ≈ 0 indicate no effects. Note the difference in the gradient scale limits.

Comparison between baseline 15 °C and warm pulse treatment revealed a “region of existence” delimited by a boundary (black isoline) above which the organisms’ performance is compromised. Survival was impacted by all warm pulse intensities with the outcome, either positive or negative, depending on the timing of the warm pulse (Fig. 5a). Intense pulses consistently resulted in lower survival compared to the constant 15 °C, regardless of the timing; low and intermediate pulses only decreased survival when they occurred after day 10, while earlier warm pulse increased survival. The threshold beyond which any warm pulse resulted in a decline in larval fitness was broader (Fig. 5b), with nearly all warm pulses leading to reduced fitness.

### Warm press experiment

**Fig. 6:**
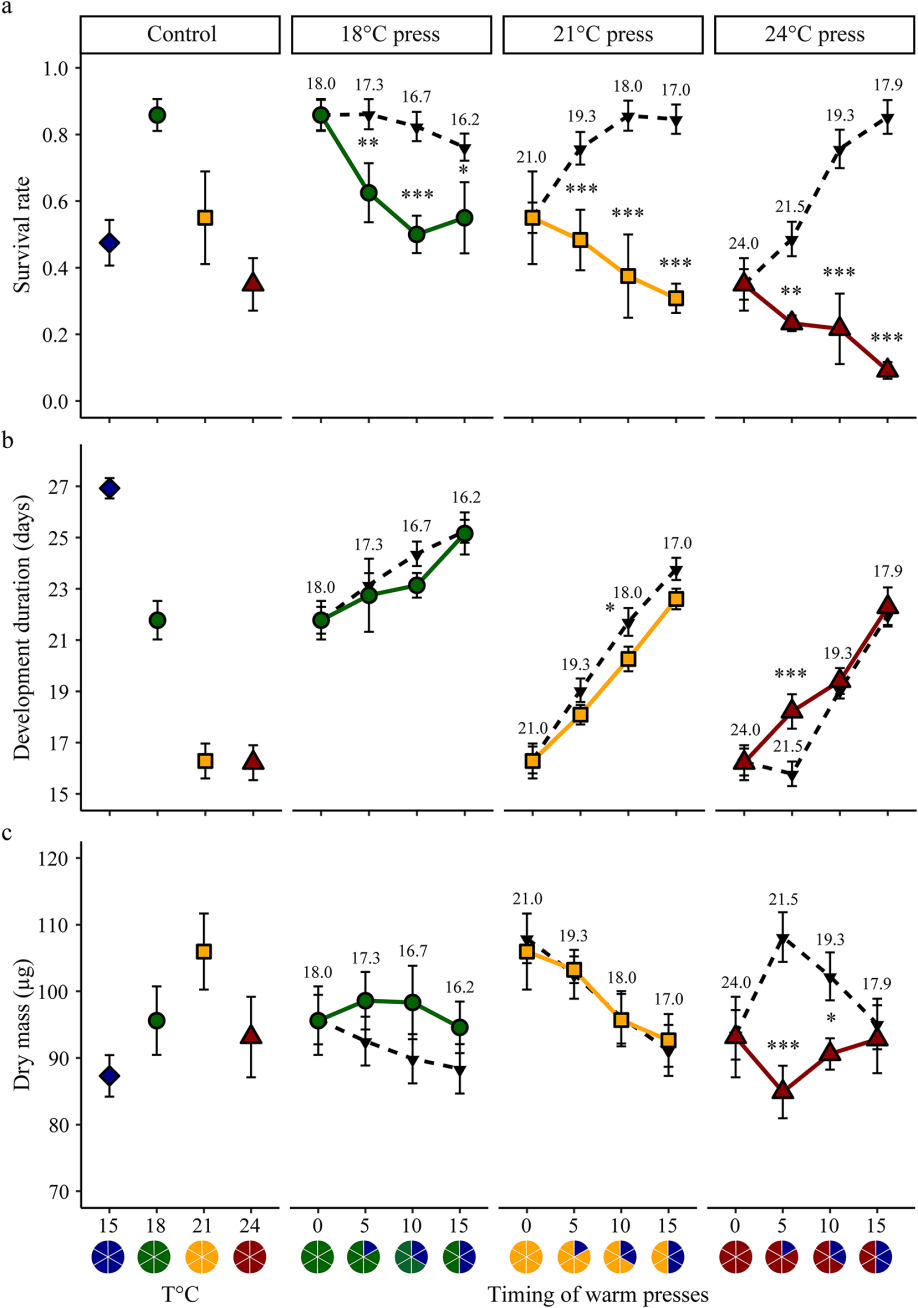
Survival rate, development duration, and dry mass after exposure to warm presses. Comparison between observed (18 °C: filled green circle; 21 °C: filled yellow rectangle ; and 24 °C: filled red triangle warm press) and expected (filled inverted triangle) values under average temperature experienced throughout the experiment. (**a**) Survival rate to megalopa, (**b**) development duration from hatching to megalopa and (**c**) megalopa dry mass reared at control constant (left panel) or under warm press (right panels). Temperatures: 15 °C filled blue diamond, 18 °C filled green circle, 21 °C filled yellow rectangle and 24 °C filled red triangle). Each point represents the mean value ± standard error for each treatment per female (*n* = 4). Values above or below the black dotted line represent the average constant temperature experienced (°C) during the warm presses. Asterisks indicate significant differences between expected and observed values for each treatment. *p* < 0.05*, *p* < 0.01**, *p* < 0.001***. Note that each timing 0 is equivalent to the controls (i.e., larvae reared at constant temperatures: 18, 21, and 24 °C from hatching until metamorphosis; left panel). Pie charts indicate control and warm press treatments.

Survival rate to megalopa decreased both with intensity (Tukey-adjusted *p* < 0.001 for all comparisons) and timing (Fig. 6a). Consistently, the biggest drops in survival rates were observed during late warm presses. For example, during a late 24 °C press, only 9% of larvae reached megalopa, compared to 35% survival during early warm presses of the same intensity. The observed survival rates were much lower than those expected from the average temperature (Fig. 6a; Table 2, S3).

**Table 2:** Warm press experiment: Best model selected for each variable of interest, based on AICc.

Variable	Best model	ΔAICc	2nd best model	Supplementary file
Survival rate	t + T°C	7	t × T°C	Table S6
Development duration	t × T°C	23	t + T°C	Table S6
Dry mass	t + T°C	6	t × T°C	Table S6
Instantaneous growth rate	t × T°C	142	t + T°C	Table S6
Predicted vs. expected survival rate	EO × T°C × t	15	EO × t + EO × T°C + T°C × t	Table S3
Expected vs. Observed development duration	EO × T°C × t	4	EO × t + EO × T°C + T°C × t	Table S4
Expected vs. Observed dry mass	EO × T°C × t	41	EO × T°C + T°C × t	Table S5
Expected vs. Observed instantaneous growth rate	EO × T°C × t	92	EO × t + EO × T°C + T°C × t	Table S5

Factors include t: warm press timing, T°C: warm press intensity, EO: Expected vs. Observed value. ΔAICc: difference between the best model and the next closest model. Complete model selection tables are available in the supplementary file.

Development duration to megalopa was significantly longer at the 18 °C press (Tukey-adjusted *p* < 0.001 compared with intermediate and higher intensities) and when it started later in the larval cycle (Tukey-adjusted *p* < 0.001 for all comparisons) (Fig. 6b). Expectations from average temperatures did not always match the observed development duration with deviation observed for the intermediate and intense presses starting at day 5 and 10 respectively (Fig. 6b; Table 2, S4).

The best models included the additive and interactive effect between intensity and timing for dry mass and instantaneous growth rates respectively (Table 2, S6). At intermediate pulses, dry mass decreased with delayed timing (Fig. 6c). However, no clear patterns were observed at other intensities. The instantaneous growth rates declined with both the intensity and timing with a sharper decrease found at intermediate and high intensities compared to the low intensity (Fig. S4). Deviations from expectations under average temperature were also observed, however, significant deviations were not always consistent. For dry mass, they were restricted to warm presses of the highest intensity (Fig. 6c), while for growth rates they were inconsistent across the different intensities (Fig. S5).

**Fig. 7:**
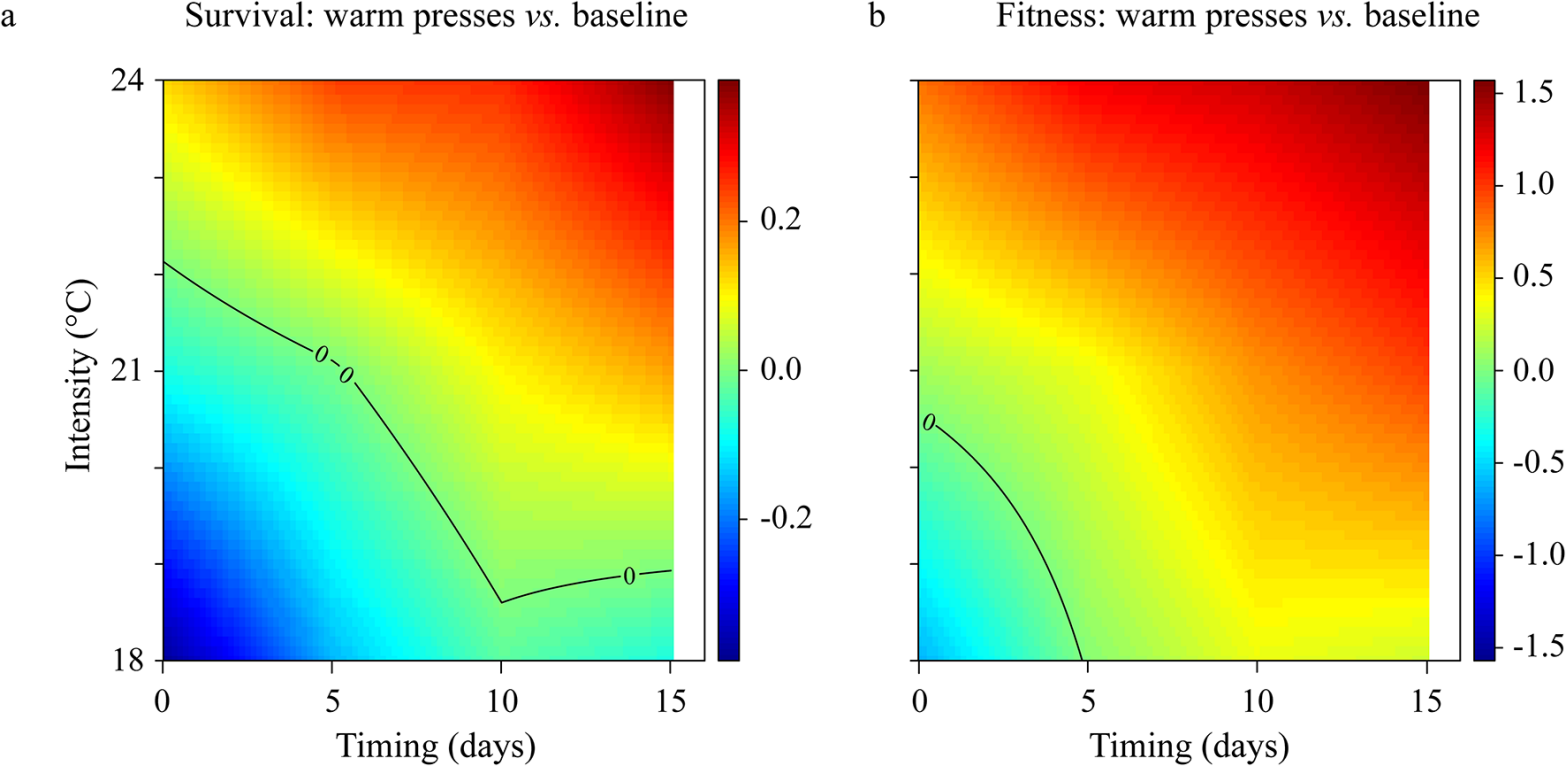
Difference in larval performance between the baseline constant temperature and warm presses condition. Differences in (**a**) larval survival and (**b**) fitness. Fitness is calculated as the total megalopa production (mg). Colour gradient represents the difference between response at the baseline temperature: 15 °C, and after exposure to warm presses. Differences < 0 indicate positive effects of the warm presses (i.e., the region below the “0” isoline). Differences > 0 indicate negative effects of the warm presses (i.e., the region extending beyond the “0” isoline). Differences ≈ 0 indicate no effects. Note the difference in the gradient scale limits.

As observed for the warm pulses, comparison between baseline 15 °C and warm press treatment showed that survival was again affected by all intensities with the effect varying depending on the timing. (Fig. 7a). Intense presses consistently led to lower survival compared to the constant 15 °C, regardless of when they occurred. The region of existence (black isoline) was limited to the bottom corner of the space of changes in timing and intensity; low intensity presses increased survival rates in comparison to those observed at constant 15 °C. Intermediate presses resulted in lower survival for all presses occurring after 5 days and higher survival for all presses occurring at t_5_ and earlier. The threshold beyond which any warm presses resulted in a decline in fitness was broader (Fig. 7b) with nearly all warm presses leading to reduced fitness, except for early ones below 21 °C.

## Discussion

We investigated the response of *C. maenas* larvae to warm events (i.e., idealized heatwaves) of different intensity, timing, and duration. Our study yielded two main findings. First, larval performance responded to the combined effects of the heatwave components, in a manner that cannot easily be disentangled. Second, our study highlighted that in several combinations of timing and intensity, biological responses under heatwaves could not be predicted by using the average temperature experienced throughout the heatwave (i.e., constant conditions).

In this study, we identified a “region of existence” defined by a boundary, beyond which the organisms’ performance becomes compromised compared to their performance at the baseline temperature of 15 °C. This region also identified the set of warm events that may be considered as “extreme” for the organism^47^. As expected, the boundary responds to the fact that higher intensities lead to stronger reductions in survival and overall fitness. Additionally, the negative effects of intensity on survival and fitness were exacerbated when the warm events occurred later in the larval phase. Indeed, this interactive effect also reaches a point where the response occurs at low intensities. The effect of timing found in this study points towards the stage-dependent costs of acute temperature exposures. These findings highlight the importance of timing in assessing organisms’ response to environmental changes, offering new insights that could reshape our understanding of the effect of heatwaves on organisms with complex life cycle such as meroplankton.

The role of timing is further highlighted by the results of the second experiment in which late presses were shorter than early ones and led to a stronger negative effect on performance. This points to the last zoeal stage and metamorphosis to megalopa as a critical bottleneck. This result is consistent with the transition timing hypothesis^65^, where physiological tolerance is reduced in stages previous to major transitions. Indeed, transitions between ontogenetic stages are particularly sensitive to environmental factors such as temperature^66^. Enhanced mortality typically occurs near the start and end of the larval phase in crustaceans^53,67,68^ and early embryos appear to be more tolerant to temperature increases than later stages in sea urchins^69^ and starfishes^70^. Furthermore, a study on copepods found that heat exposure during the naupliar stage had no effect on later stages, suggesting resilience to sub-lethal but intense heat exposure^71^. Similarly, research on terrestrial organisms showed that insects exposed to high temperature stress during early-stage can recover from it if subsequent stages are not stressed. In contrast, temperature increase during later stages often leads to irreversible damage^72–74^. Therefore, our results highlight a need to explore the consequences of a match/mismatch between the timing of a heatwave and that of metamorphosis in species with complex life cycles.

The effect of the heatwave components on the space of existence is relevant to understand heatwave effects in nature. For any species, the boundary of the space of existence can be used to define the group of event experienced as “extreme”^47^, defined as “an episode where climate or climate-driven conditions trigger a negative threshold-like biological response”^75^. When such extreme events differ in timing and intensity, they are likely to differ from the event defined in the literature as heatwaves. For example, marine heatwaves are usually defined through climatological thresholds, as events “lasting for five days or more, with temperatures exceeding the threshold of 90th percentile of the historical baseline”^16^. The 90th percentile temperature is not constant but fluctuates with the seasons. Indeed, from spring to summer, when larvae of *C. maenas* develop, the 90th percentile increases^48^. Hence, late heatwaves (i.e., summer) will be associated with higher thermal thresholds than early ones (i.e., spring). Our findings revealed that, as development progresses, the boundary of the space of existence was reduced to lower intensities. Given the high threshold, it is likely that acute temperature exposures in summer below the threshold (therefore not classified as heatwaves) will still be enough to depress survival. In contrast, earlier in the season, during periods when the threshold is lower, acute temperature exposures may be classified as heatwaves, though they will not always negatively affect survival. Therefore, depending on the timing, some heatwaves may not always trigger biological responses^75^, while other warm events not classified as heatwaves could still have significant impacts^76^. This emphasizes the need to assess heatwaves on a seasonal basis when evaluating biological responses. This statement corroborates with other findings on a wide range of marine organisms such as fish that showed a bigger sensitivity to winter marine heatwaves than summer ones despite the latter having higher temperatures^18^, or copepods that can exhibit strong seasonal variation in their vulnerability to heatwaves depending on the species studied^19^. In another study, authors found that diatoms growing in warmer temperatures prior to a heatwave experienced even greater negative effects than those growing in cooler conditions^17^, further emphasizing the crucial role of season in shaping heatwave impacts.

**Fig. 8:**
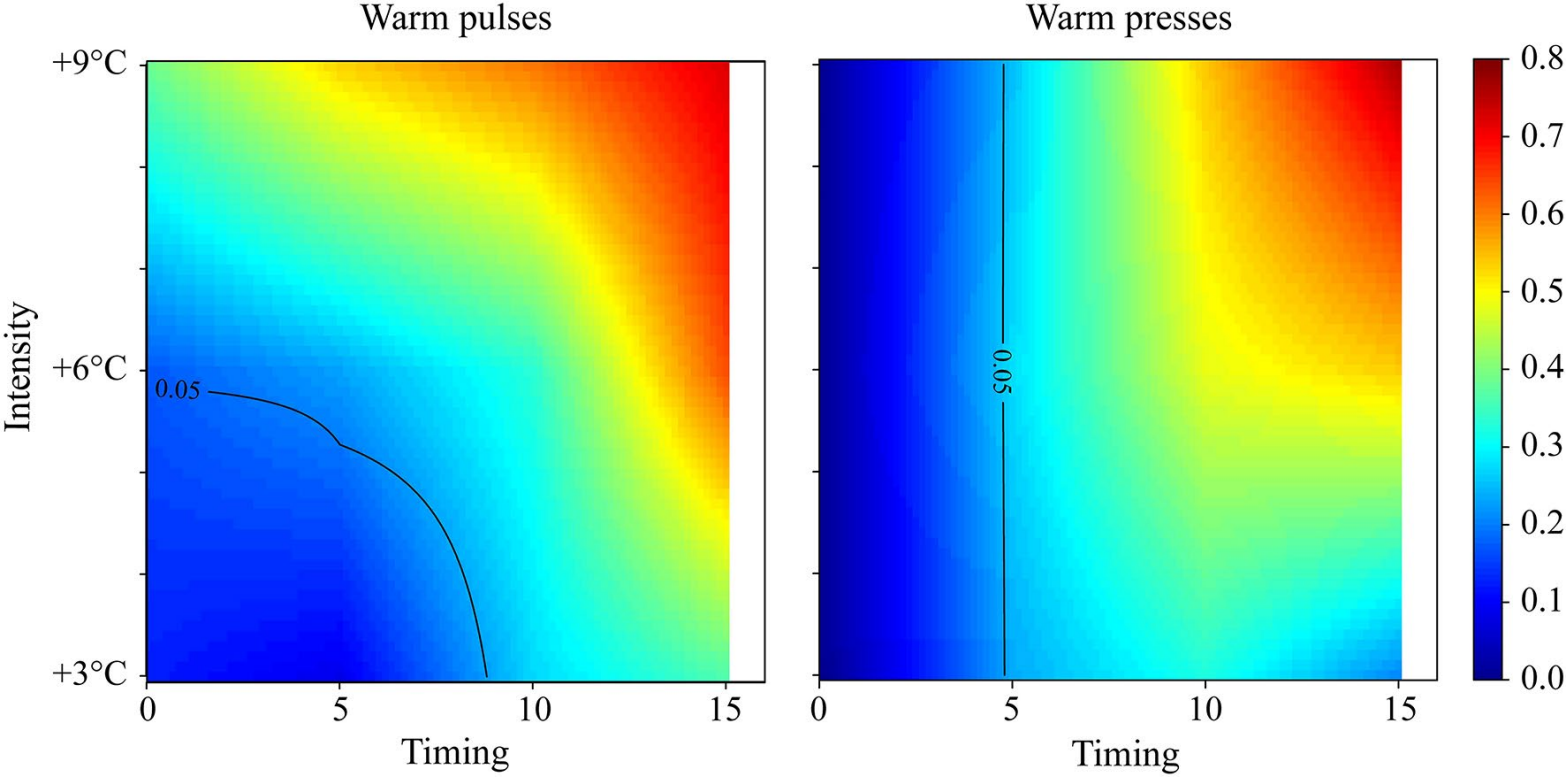
Difference between the observed survival during warm pulses and presses and the expectation for the average temperature experienced throughout the experiment (color gradient). Intensity (left axis) is shown as °C (+ 3 for 18 °C, + 6 for 21 °C, and + 9 for 24 °C). The black line represents the threshold of statistical significance (*p* = 0.05).

We identified a region in the space of changes in components where biological responses cannot be predicted by the average temperature experienced over the developmental period, specifically regarding the survival (Fig. 8). This discrepancy is significant for two reasons: first, it points towards the potential failure of a mechanism or the triggering of a compensatory response, not observed under constant temperatures. Second, it helps to identify scenarios where responses to heatwaves can be predicted from simple experiments, keeping organisms under constant conditions. Here, we found that regardless of the timing, under intermediate and intense pulse, constant temperature failed to predict the responses. This failure was restricted to late timing during low-intensity pulses. Interestingly the discrepancy between observed and expected values was not always consistent. Notably, the greater the intensity and/or the later the timing, the larger the difference tended to be. Concerning the warm presses, when the event occurred between 5 days after hatching onwards, prediction failures occurred regardless of the intensity. The differences between the two experiments suggest a role for recovery time post-heatwave if such heatwaves occurred between days 5 and 10.

Another important finding was that the strongest deviations from expectations made from the average temperature occurred in survival, while developmental time and megalopa body mass in response to warm events did not differ as much from expectations. It is expected that survivors will be those with robust physiological compensatory mechanisms to handle warm events. However, it is less evident that such mechanisms result in responses that do not differ from expectations under constant temperatures. Theory suggests that thermal fluctuations should lead to costs, such as prolonged development or reduced growth^77^. However, in our study, these costs appear high enough to impact survival.

Temperature fluctuations in a given environment can influence biological systems through mechanisms distinct from those at play in constant environments^44^. This discrepancy can be explained by Jensen’s inequality, originating in nonlinear relationships between temperature and biological responses^78,79^. For instance, studies have shown that constant temperatures do not accurately predict the performance of an ectotherm organism subject to fluctuating temperatures^42,43^. Hence, some mechanisms cannot be explained when the non-linearity is taken into account. Indeed, during a heatwave, the temperature might surpass some thermal threshold, triggering irreversible physiological damage or leading to physiological perturbations that require long recovery times. Such phenomena would not occur if individuals are exposed to low average temperatures over a long time. Conversely, matches between responses to constant temperature vs. warm event would occur if temperature operates cumulatively on developmental processes.

An important question from this study concerns how realistic heatwaves drive larval performance. While our results can be used for prediction, they can also guide for future research, keeping in mind that daily temperature changes under natural conditions occur much slower than those used in our experiments. For instance, our findings suggest that the temperature of spring heatwaves in the German Bight (i.e., maximum temperature ranging from 6.1 °C to 16.3 °C:^48^) does not reach the level required to negatively affect performance, even considering the abrupt temperature change experienced in the laboratory. Additionally, the effects of spring heatwaves could be accurately estimated using expectations from experiments with constant temperatures. However, the effect of summer heatwaves on larval survival may not be well-predicted if they occur when larvae are at advanced stages of development.

While we did not manipulate heatwave components such as the rate of temperature increase, our study can serve as an initial exploration of the space of heatwave components. Indeed, with a temperature increase rate set to a constant and high value, we found that early warm events only affected larval performance during high intensity. Based on the assumption that realistic heatwaves have lower rates of temperature increase, providing larvae with sufficient time to acclimate, we can hypothesize that early heatwaves are unlikely to impact survival. This result suggests the importance of focusing on heatwaves occurring later in the larval cycle. It will be interesting in future experiments to investigate the effect of varying temperature increase rates, combined with intensity, in order to simulate more complex and realistic heatwave scenarios.

In our study, the size and complexity of the experiment, as well as the length of the larval season, led to limitations in the number of females used to replicate the experiment. For example, conducting simultaneous experiments with larvae from different berried females was not possible. We found, however, that responses to constant temperature were consistent with those reported for the local population in previous studies^50,80–82^. It is important to note that variation in larval responses may reflect genetic variation and parental effects^83^. Genetic studies on *C. maenas* in Europe suggest high gene flow across the North Sea^84^, while numerical models of larval dispersal indicate strong connectivity across the German Bight^85^. This suggests that larvae from females in the North Sea share a similar response. Additionally, previous studies demonstrate that exposure to low salinity during embryonic development (such as in Baltic Sea populations) significantly influences larval performance^81^. Therefore, our findings should be applicable to the North Sea population, as long as the embryos experience environmental conditions similar to those of our study. Moreover, while thermal tolerance patterns appear relatively consistent among populations from comparable latitudes and salinity conditions (e.g., USA^86^), future studies are needed to identify which local populations may be particularly sensitive to different heatwave components. For instance, experiments on larvae from the Cadiz population reveal slightly better performance at 24 °C than at 15 °C, suggesting distinct responses to heatwave intensity and duration^80^.

## Conclusion

In this study, we demonstrate that the timing, intensity, and duration of heatwaves can influence biological performance, resulting in varied larval responses depending on the scale of the heatwave components. We have also identified a “region of existence” where warm pulses are not considered extreme events for the organisms. This region’s boundaries may vary, narrowing or expanding based on the organisms’ traits. An expansion of this region would likely benefit species that are adapted to warmer conditions, potentially facilitating their spread and encouraging ecological invasions. By considering the dynamics of heatwaves rather than constant conditions, we can determine more precisely how organisms could respond under realistic scenarios. This understanding is crucial for informing conservation strategies for species that have complex life cycles and are highly sensitive to temperature changes.

## Electronic supplementary material

Below is the link to the electronic supplementary material.

Supplementary Material 1

## Data Availability

The data supporting the results of this study will be made available in the PANGAEA public repository, upon acceptance of the manuscript.
