# Supplementary material for: Heatwave duration, intensity and timing as drivers of performance in larvae of a marine invertebrate
Source: Sci Rep. 2025 May 7;15:15949. doi: 10.1038/s41598-025-98259-7 (PMC12059064; doi:10.1038/s41598-025-98259-7)
Supplement: Supplementary file 1 — Supplementary Material 1 [file 41598_2025_98259_MOESM1_ESM.docx]

# Supplemental information

*Section I: Before effects*

An important point in the analysis was to determine if any effects of the treatments characterising warm pulses and presses were observed before experiencing the warm events. We checked for these effects by looking at performance at the second, third and fourth zoeal stages. The interaction between intensity and timing was retained in the model for development to zoea II, III and IV during warm pulses and presses (Table S1).

**Table S1.** Model selection based on AICc for development duration at zoea II (ZI), zoea III (ZIII) and zoea IV (ZIV) in response to warm pulses and presses timing (t) and intensity (T°C). Results are shown for linear and logarithmic scales. Model selection was performed using Restricted Maximum Likelihood (REML) and Maximum Likelihood (ML), for random and fixed factors respectively. The best models are indicated in bold. Red indicates violation of model assumptions. Female of origin (♀) was always included in the model as a random factor.

|  |  | **Warm pulses** | | | | | | | | | | **Warm presses** | | | | | | |
| --- | --- | --- | --- | --- | --- | --- | --- | --- | --- | --- | --- | --- | --- | --- | --- | --- | --- | --- |
| **Model selection** | **Linear scale** | | | | **Logarithmic scale** | | | | | | | **Linear scale** | | | | **Logarithmic scale** | | |
|  | **ZII** | | **ZIII** | **ZIV** | | **ZII** | **ZIII** | **ZIV** | | **ZII** | | | **ZIII** | **ZIV** | **ZII** | | **ZIII** | **ZIV** |
| ***Random factors (REML)*** | | | | | | | | | | | | | | | | | | |
| t × T°C \| ♀ | 605 | | 289 | 391 | | -274 | -339 | -319 | | 232 | | | 329 | 316 | -254 | | -311 | -389 |
| T°C \| ♀ | 230 | | 288 | 377 | | -249 | -341 | -327 | | 233 | | | 330 | 308 | -250 | | -300 | -384 |
| t \| ♀ | **181** | | 296 | 376 | | **-302** | -336 | **-340** | | **214** | | | **308** | **297** | **-278** | | **-321** | **-413** |
| 1 \| ♀ | 220 | | **286** | **373** | | -260 | **-345** | -335 | | 222 | | | 326 | 299 | -236 | | -309 | -394 |
| ***Fixed factors (ML)*** | | | | | | | | | | | | | | | | | | |
| t × T°C | **153** | | **267** | **362** | | **-375** | **-423** | | **-418** | | **188** | | **310** | **278** | **-348** | | **-386** | **-497** |
| t + T°C | 180 | | 312 | 406 | | -326 | -350 | | -343 | | 214 | | 357 | 372 | -309 | | -302 | -372 |
| T°C | 189 | | 606 | 623 | | -317 | -65 | | -335 | | 223 | | 585 | 382 | -302 | | -296 | -364 |
| t | 211 | | 383 | 520 | | -293 | -277 | | -227 | | 228 | | 385 | 495 | -292 | | -268 | -257 |

No significant variations in development were observed for zoea II and III at t_10_ and t_15_ across all intensities (Fig. S1-S2). Moreover, development duration at a constant temperature of 15 °C was similar to those observed after t_10_ and t_15_ warm events (Fig. S1-S2). This lack of difference can be attributed to a “before” effect as the larvae were moulting to zoea III one day prior to the onset of the warm event, hence remaining under the baseline temperature conditions. The t_10_ observation is attributed to a “start” effect as the larvae experienced the warm pulses for two days only before reaching zoea III. Similar patterns emerged for zoea IV where no significant differences in development were detected at t_15_ between 21 and 24 °C warm events and development duration were similar than under 15 °C constant condition. Given that larvae experienced the warm events for only 2.5 days, the development under 21 and 24 °C warm events at t_15_ can be attributed to a “start” effect.


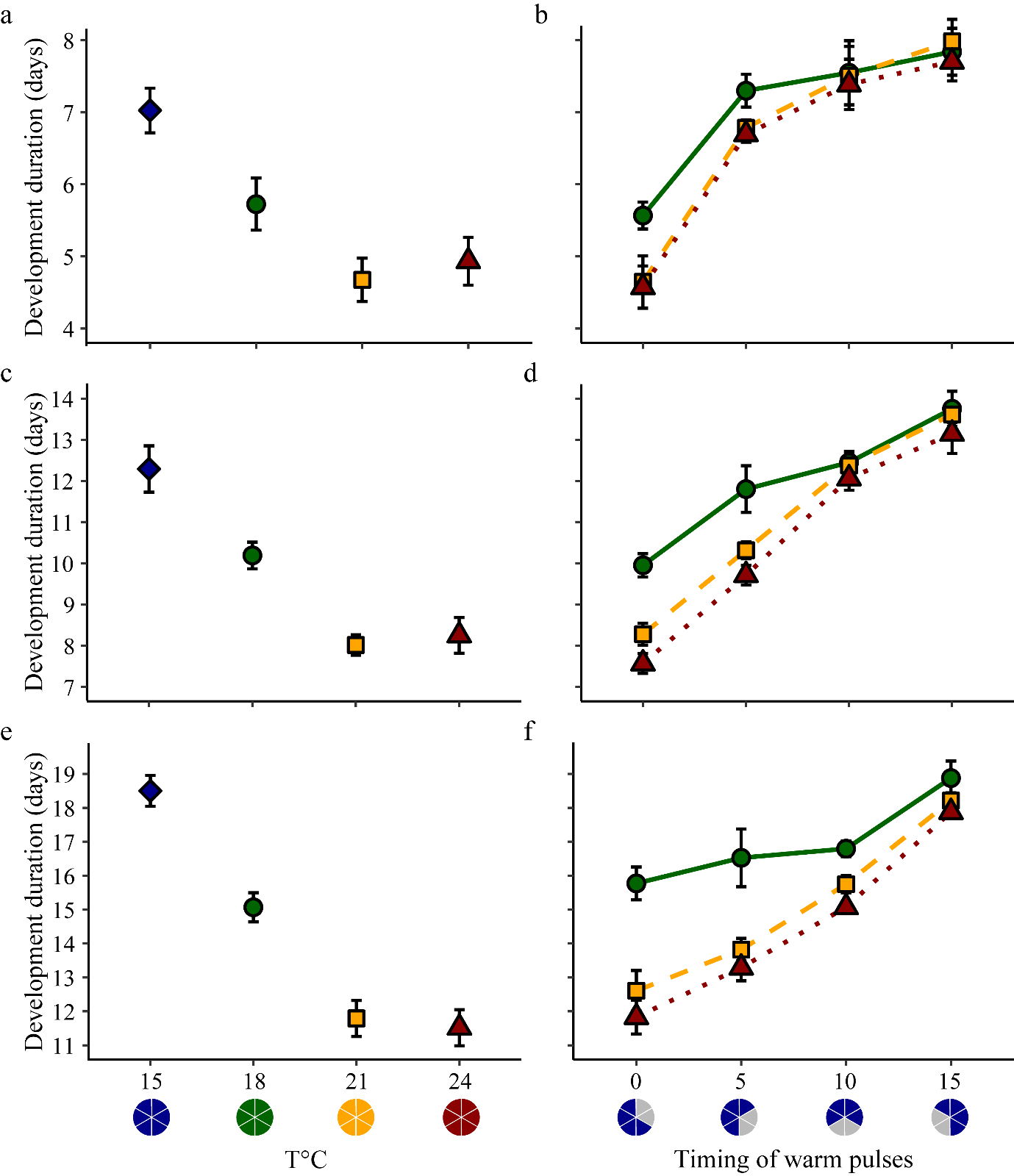
**Figure S1.** Duration of larval development from hatching until **a.** zoea II, **b.** zoea III and **c.** zoea IV. in response to constant temperature (control; left panels) and in response to warm pulses at different timing and temperature (right panels). Values shown are the mean ± standard error for each treatment among the four females of origin. Temperatures: 15°C:
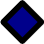
 18°C:
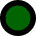
 21°C:
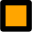
 24°C:
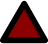
. The grey sections of the pie represent the timing of each warm pulses.

**
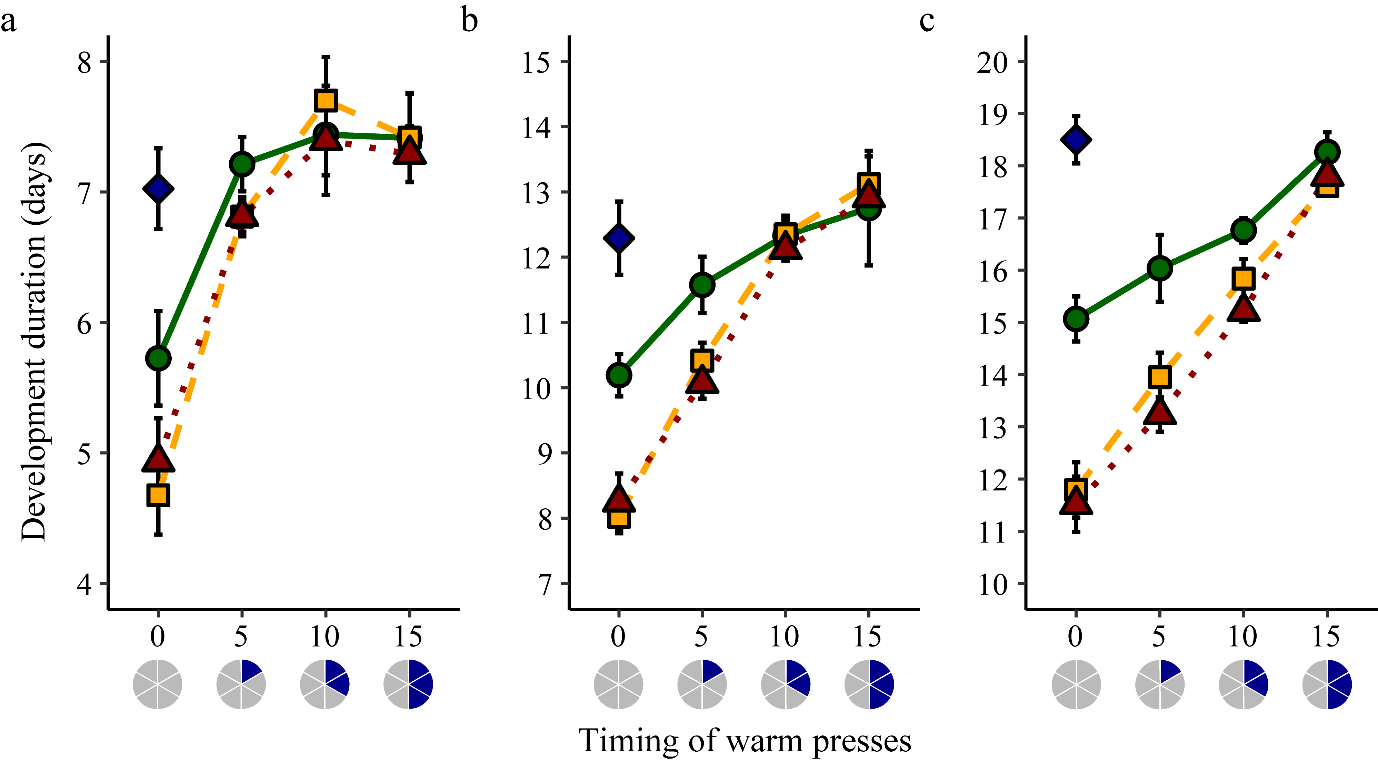
Figure S2.** Development duration from hatching until **a.** zoea II, **b.** zoea III and **c.** zoea IV in response to warm presses at different timing and temperature. Values shown are the mean ± standard error for each treatment among the four females of origin. Temperature: 15°C:
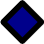
 18°C:
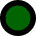
 21°C:
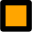
 24°C:
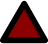
. The grey sections of the pie represent the timing of each warm pulses.

*Section II: Post heatwaves effects*

**Table S2.** Model selection based on AICc for development duration until megalopa, survival from hatching to megalopa, megalopa dry mass and instantaneous growth rate from hatching to megalopa in response to warm pulses timing (t) and intensity (T °C). Results are shown for linear and logistic for survival, linear and logarithmic scales for development, and linear for dry mass and instantaneous growth rate. Model selection was performed using Restricted Maximum Likelihood (REML) and Maximum Likelihood (ML), for random and fixed factors respectively. The bests models are indicated in bold. Red indicates violation of model assumptions. Female of origin (♀) was always included in the model as a random factor.

|  | **Survival rate** | | **Development duration**  **(days)** | | **Dry mass (µg × ind^-1^)** | **Instantaneous growth rate (day^-1^)** |
| --- | --- | --- | --- | --- | --- | --- |
| **Model selection** | **Linear scale** | **Logistic scale** | **Linear scale** | **Logarithmic scale** | **Linear** | **Linear** |
| ***Random factors (REML)*** | | | | | | |
| t × T°C \| ♀ | 34 | 447 | 558 | -209 | 5024 | -3230 |
| T°C \| ♀ | 15 | 430 | 545 | -221 | 4887 | -3369 |
| t \| ♀ | 25 | 440 | 555 | -213 | 4883 | -3361 |
| 1 \| ♀ | **6** | **420** | **536** | **-232** | **4881** | **-3370** |
| ***Fixed factors (ML)*** | | | | | | |
| t × T°C | -40 | 412 | 543 | -301 | 4924 | **-3495** |
| t + T°C | **-42** | **407** | **535** | **-307** | **4918** | -3495 |
| T°C | 6 | 450 | 566 | -275 | 4926 | -3466 |
| t | 1 | 450 | 626 | -209 | 4942 | -3464 |

**Table S3.** Model selection based on AICc for proportion of survival. Comparison between expected and observed (EO) survival to megalopa in response to warm pulses and presses timing (t) and intensity (T°C). Results are shown for linear and logistic scales. Female of origin (♀) was always included in the model as a random factor. Model selection was performed using Restricted Maximum Likelihood (REML) and Maximum Likelihood (ML), for random and fixed factors respectively. The best models are indicated in bold. Red indicates violation of model assumptions. Female of origin (♀) was always included in the model as a random factor.

|  |  | **Warm pulse** | | | **Warm press** | |
| --- | --- | --- | --- | --- | --- | --- |
|  | **Model selection** | **Linear scale** | **Logistic scale** | | **Linear scale** | **Logistic scale** |
| **T°C prediction** | ***Random factors (REML)*** | | | | | |
|  | T°C \| ♀ | 36 | | 179 | 36 | 179 |
|  | 1 \| ♀ | **34** | | **176** | **34** | **176** |
|  | ***Random factors (REML)*** | | | | | |
|  | t × T°C \| ♀ | -502 | 311 | | 662 | 662 |
|  | T°C \| ♀ | **-519** | **294** | | 651 | 651 |
|  | t \| ♀ | -507 | 306 | | 722 | 722 |
|  | 1 \| ♀ | **-514** | **298** | | **641** | **641** |
|  | ***Fixed factors (ML)*** | | | | | |
|  | **Three-way** | | | | | |
|  | EO × T°C × t | -656 | 234 | | **-259** | **609** |
|  | **3 two-way** | | | | | |
|  | EO × t + EO × T°C + T°C × t | **-661** | **226** | | -244 | 629 |
|  | **2 two-way** | | | | | |
|  | EO × T°C + EO × t | -642 | 245 | | -212 | 660 |
|  | EO × T°C + T°C × t | -610 | 272 | | -144 | 730 |
|  | EO × t + T°C × t | -604 | 282 | | -227 | 641 |
|  | **Two-way** | | | | | |
|  | EO + T°C × t | -569 | 313 | | -135 | 736 |
|  | EO × T°C + t | -592 | 290 | | -128 | 744 |
|  | EO × t + T°C | -586 | 299 | | -199 | 669 |
|  | **Additive** | | | | | |
|  | EO + T°C + t | -552 | 330 | | -121 | 749 |
|  | EO + T°C | -556 | 325 | | -126 | 743 |
|  | EO + t | -302 | 568 | | -10 | 861 |
|  | T°C + t | -405 | 478 | | 5 | 880 |
|  | T°C | -409 | 475 | | 89 | 874 |
|  | t | -150 | 722 | | -15 | 958 |
|  | EO | -306 | 653 | | -26 | 855 |

**Table S4.** Model selection based on AICc for development duration. Comparison between expected and observed (EO) development to megalopa in response to warm pulses and press timing (t) and intensity (T°C). Results are shown for linear and logarithmic scales. Female of origin (♀) was always included in the model as a random factor. Model selection was performed using Restricted Maximum Likelihood (REML) and Maximum Likelihood (ML), for random and fixed factors respectively. The best models are indicated in bold. Female of origin (♀) was always included in the model as a random factor.

|  |  | **Warm pulse** | | **Warm press** | |
| --- | --- | --- | --- | --- | --- |
|  | **Model selection** | **Linear scale** | **Logarithmic scales** | **Linear scale** | **Logarithmic scales** |
| **T°C prediction** | ***Random factors (REML)*** | | | | |
|  | T°C \| ♀ | 192 | -65 | 192 | -65 |
|  | 1 \| ♀ | **187** | **-66** | **187** | **-66** |
|  | ***Random factors (REML)*** | | | | |
|  | t × T°C \| ♀ | 695 | -801 | 644 | -838 |
|  | T°C \| ♀ | 686 | -810 | 641 | -841 |
|  | t \| ♀ | 688 | -808 | 645 | -837 |
|  | 1 \| ♀ | **681** | **-815** | **639** | **-842** |
|  | ***Fixed factors (ML)*** | | | | |
|  | **Three-way** | | | | |
|  | EO × T°C × t | 659 | -986 | **612** | **-1017** |
|  | **3 two-way** | | | | |
|  | EO × t + EO × T°C + T°C × t | **647** | **-997** | 616 | -1003 |
|  | **2 two-way** | | | | |
|  | EO × T°C + EO × t | 650 | -992 | 865 | -719 |
|  | EO × T°C + T°C × t | 667 | -979 | 619 | -999 |
|  | EO × t + T°C × t | 662 | -978 | 640 | -970 |
|  | **Two-way** | | | | |
|  | EO + T°C × t | 679 | -963 | 642 | -967 |
|  | EO × T°C + t | 670 | -975 | 866 | -718 |
|  | EO × t + T°C | 666 | -974 | 884 | -696 |
|  | **Additive** | | | | |
|  | EO + T°C + t | 682 | -960 | 885 | -696 |
|  | EO + T°C | 722 | -920 | 1237 | -355 |
|  | EO + t | 1232 | -420 | 1254 | -344 |
|  | T°C + t | 698 | -929 | 884 | -696 |
|  | T°C | 736 | -893 | 1235 | -356 |
|  | t | 1237 | -410 | 1252 | -346 |
|  | EO | 1241 | -410 | 1389 | -206 |

*
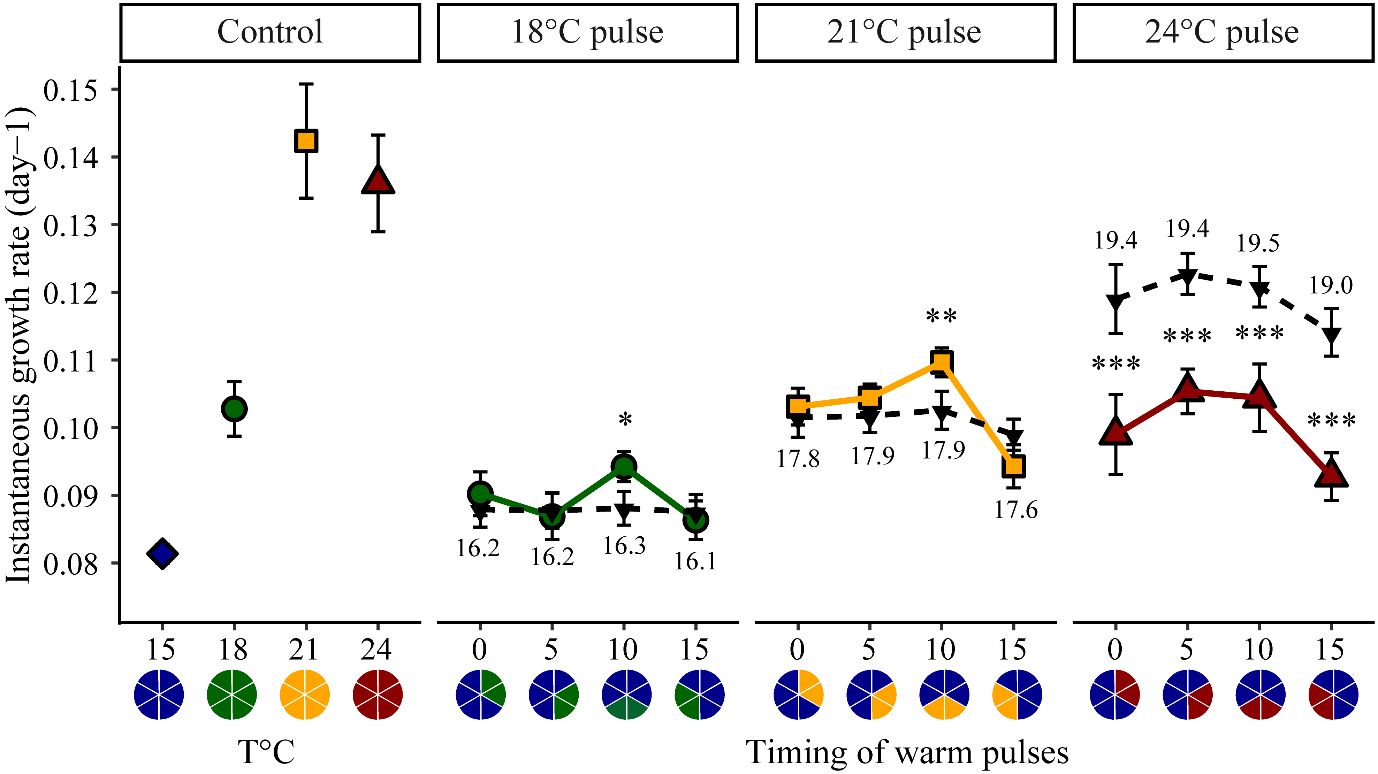
***Figure. S3.** Instantaneous growth rate during warm pulses. Comparison between observed (18 °C:
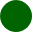
 ; 21 °C:
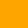
 ; 24 °C:
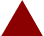
) and expected (
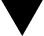
) growth rates for larvae reared at constant (control: left panel) or une warm pulses (right panels). Temperature: 18 °C
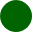
, 21 °C
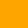
 and 24 °C
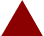
. Each point represents the mean value ± standard error for each treatment per female (n = 4). Values above or below the black dotted line represent the mean constant temperature experienced (°C) during the warm pulses. Asterisks indicate significant differences between expected and observed values for each treatment. *p* < 0.05*. *p* < 0.01**, *p* < 0.001***. Pie charts indicate control and warm pulse treatments.

**Table S5.** Model selection based on AICc for growth traits comparison between expected and observed value (EO) in response to warm pulses and presses timing (t) and intensity (T°C). Results are shown for linear and logarithmic scales. Female (♀) was always included in the model as a random factor. Model selection was performed using Restricted Maximum Likelihood (REML) and Maximum Likelihood (ML), for random and fixed factors respectively. The best models are indicated in bold. Female of origin (♀) was always included in the model as a random factor.

|  |  | **Warm pulse** | | **Warm press** | |
| --- | --- | --- | --- | --- | --- |
|  | **Model selection** | **Dry mass (µg × ind^-1^)** | **Instantaneous growth rate (day^-1^)** | **Dry mass (µg × ind^-1^)** | **Instantaneous growth rate (day^-1^)** |
| **Model for T°C prediction** | ***Random factors (REML)*** | | | | |
|  | T°C \| ♀ | 2 step-approach | **-327** | 2 step-approach | 2 step-approach |
|  | 1 \| ♀ |  | -297 |  |  |
|  | ***Random factors (REML)*** | | | | |
|  | t × T°C \| ♀ | 6826 | -8262 | **6245** | **-9683** |
|  | T°C \| ♀ | 6815 | **-8262** | 6271 | -8726 |
|  | t \| ♀ | 6833 | -8232 | 6264 | -8677 |
|  | 1 \| ♀ | **6827** | -8235 | 6268 | -8421 |
|  | ***Fixed factors (ML)*** | | | | |
|  | **Three-way** | | | | |
|  | EO × T°C × t | 6849 | -8534 | **6266** | **-9935** |
|  | **3 two-way** | | | | |
|  | EO × t + EO × T°C + T°C × t | **6843** | **-8539** | 6308 | -9843 |
|  | **2 two-way** | | | | |
|  | EO × T°C + EO × t | 6859 | -8537 | 6446 | -9789 |
|  | EO × T°C + T°C × t | 6848 | -8526 | 6307 | -9838 |
|  | EO × t + T°C × t | 6944 | -8368 | 6364 | -9763 |
|  | **Two-way** | | | | |
|  | EO + T°C × t | 6950 | -8356 | 6366 | -9760 |
|  | EO × T°C + t | 6864 | -8524 | 6446 | -9784 |
|  | EO × t + T°C | 6960 | -8365 | 6503 | -9709 |
|  | **Additive** | | | | |
|  | EO + T°C + t | 6970 | -8353 | 6504 | -9706 |
|  | EO + T°C | 6996 | -8353 | 6577 | -9684 |
|  | EO + t | 8525 | -8317 | 6559 | -9685 |
|  | T°C + t | 6976 | -8345 | 6504 | -9701 |
|  | T°C | 7007 | -8345 | 6577 | -9679 |
|  | t | 8536 | -8309 | 6560 | -9680 |
|  | EO | 8531 | -8318 | 6634 | -9662 |

**Table S6.** Model selection based on AICc for survival from hatching to megalopa, development duration until megalopa, megalopa dry mass and instantaneous dry mass growth rate in response to warm presses timing (t) and intensity (T°C). Results are shown for linear and logistic for survival, linear and logarithmic scales for development, and linear for dry mass and instantaneous dry mass growth rate. Model selection was performed using Restricted Maximum Likelihood (REML) and Maximum Likelihood (ML), for random and fixed factors respectively. The best models are indicated in bold. Red indicates violation of model assumptions. Female of origin (♀) was always included in the model as a random factor.

|  | **Survival rate** | | **Development duration**  **(days)** | | **Dry mass (µg × ind^-1^)** | **Instantaneous growth rate (day^-1^)** |
| --- | --- | --- | --- | --- | --- | --- |
| **Model selection** | **Linear scale** | **Logarithmic scale** | **Linear scale** | **Logarithmic scale** | **Linear** | **Linear** |
| ***Random factors (REML)*** | | | | | | |
| t × T°C \| ♀ | 23 |  | 523 |  | 5024 | -3577 |
| T°C \| ♀ | 9 | 428 | 499 | -252 | 4887 | -3565 |
| t \| ♀ | 24 | 438 | 514 | -246 | 4883 | **-3578** |
| 1 \| ♀ | **5** | **419** | **494** | **-262** | **4881** | -3569 |
| ***Fixed factors (ML)*** | | | | | | |
| t × T°C | -41 | 411 | **498** | **-333** | 4924 | **-3703** |
| t + T°C | **-49** | **404** | 504 | -310 | **4918** | -3561 |
| T°C | -18 | 434 | 629 | -174 | 4926 | -3555 |
| t | 31 | 481 | 635 | -174 | 4942 | -3208 |


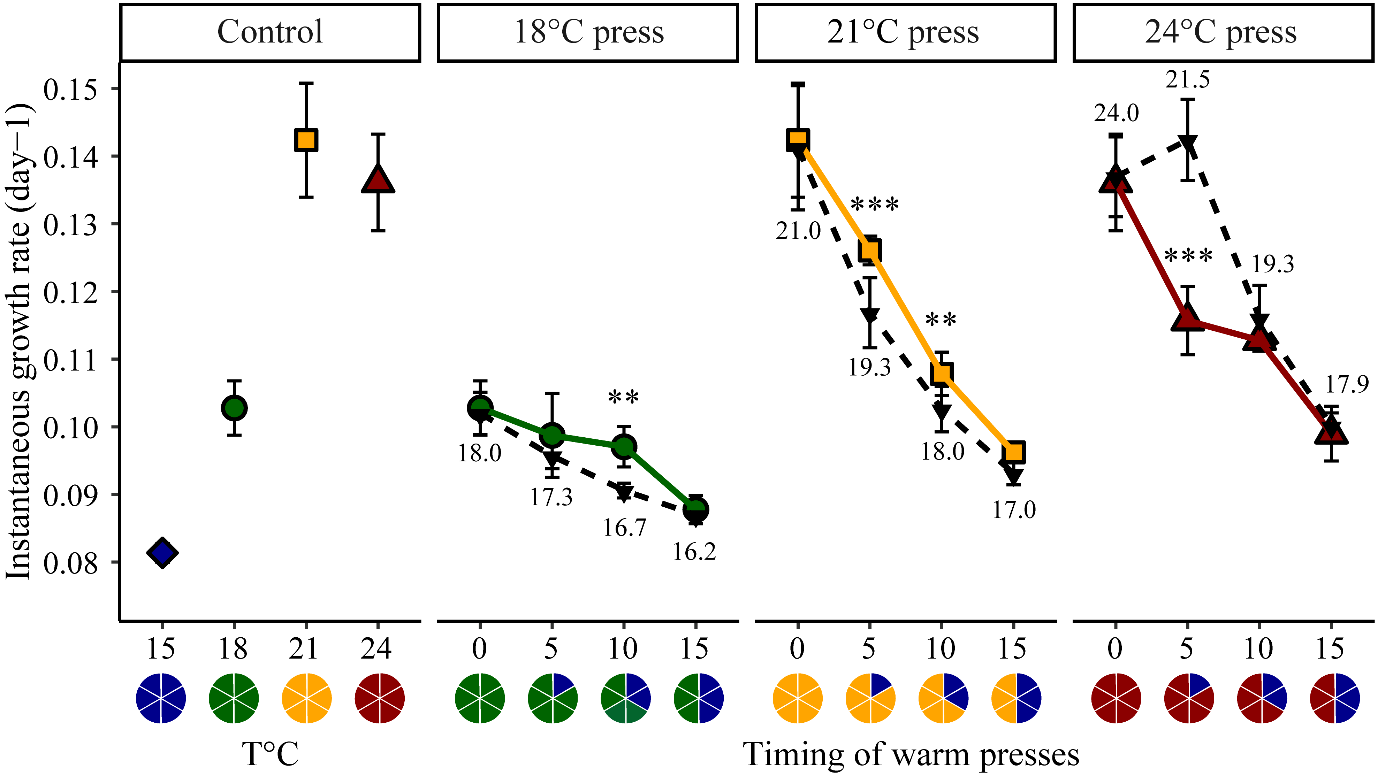
**Figure. S4.** Instantaneous dry mass growth rate during warm presses. Comparison between observed and expected (
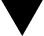
) growth rate from hatching to megalopa under 18°C (
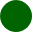
), 21°C (
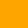
) and 24°C (
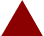
) constant temperature. Each point represents the mean value ± standard error for each treatment per female (n = 4). Values above or below the black dotted line represent the mean constant temperature experienced (°C) during the warm presses. Note that each timing 0 corresponds to the control treatments (see left panels). Pie charts indicate control and warm press treatments. Non-significant effect at t_0_ validates the accuracy of the model in predicting growth rate.
